# Effect of Letrozole Administration on Reproductive Performance and Plasma Metabolites of Ewes During Estrus Synchronization Treatment

**DOI:** 10.3390/life16071058

**Published:** 2026-06-25

**Authors:** Tingting Li, Xihu Wang, Hao Lu, Tingting Lu, Reyimu Reyilaguli, Haibo Lv, Xiaojun Liu, Jianjun Zhang, Shijie Li, Rui Xiao, Guodong Zhao

**Affiliations:** 1 Xinjiang Herbivore Nutrition Laboratory for Meat & Milk, College of Animal Science, Xinjiang Agricultural University, Urumqi 830052, China; litingting_2025@163.com (T.L.); 15569593918@163.com (H.L.); lutt11071024@163.com (T.L.); 2 Xinjiang Hutubi Breeding Cattle Farm Co., Ltd., Changji 831203, China; 18935737229@163.com (X.W.); 18997546168@163.com (X.L.);; 3 Huishang Ecological Animal Husbandry Co., Ltd., Turpan 838100, China; reylagul91@yeah.net (R.R.); 17799707298@163.com (H.L.); 4 Xinjiang Animal Embryo Engineering Technology Research Center, Changji 831203, China

**Keywords:** Turpan Black sheep, letrozole, reproductive hormones, plasma metabolites, reproductive performance

## Abstract

This study investigated the effects of letrozole (LE) on reproductive performance, hormones, and plasma metabolites in Turpan Black ewes. Sixty-six multiparous non-pregnant ewes were randomly assigned to a Control group or an LE group (0.2 mg/kg body weight, added to the basal diet) for 180 days, both receiving estrus synchronization. LE significantly increased the twinning rate (*p* < 0.05), but no significant differences were observed in estrus rate, conception rate, or lambing rate (*p* > 0.05). Hormone analysis revealed significant changes in Luteinizing Hormone (LH), Progesterone (P_4_) (group, time, and interaction effects), Estradiol (E_2_), Testosterone (T) (time effect), Gonadotropin-Releasing Hormone (GnRH) (group and interaction effects), and Follicle-Stimulating Hormone (FSH) (primarily time effect). Metabolomic analysis identified 3451 differential metabolites. L-saccharopine and 5-hydroxylysine were downregulated (*p* < 0.01), and estrone was decreased (*p* < 0.05). Lysylhistidine was upregulated (*p* < 0.05), while testosterone and LE showed rising trends without statistical significance (*p* > 0.05). These metabolites were mainly enriched in amino acid metabolism and lipid metabolism pathways related to reproduction. Significant correlations were also detected between several metabolites and reproductive hormones. LE improves the twinning rate in Turpan Black ewes, likely by modulating key reproductive hormones (LH, P_4_, GnRH, FSH, E_2_, T) and altering plasma metabolites involved in amino acid and lipid metabolism. These findings provide new insights into the regulatory mechanisms of letrozole on ovine reproduction.

## 1. Introduction

Turpan Black sheep (also known as Toksun black sheep) is a typical local dual-purpose breed for meat and fat production. It was originally derived from the crossbreeding of Bayanbulak fat-tailed sheep and Kazakh sheep [1]. Following centuries of artificial introduction, crossbreeding, and long-term targeted selection by local farmers and herdsmen, this breed has been genetically stabilized and formally established [2]. The natural twinning rate of this breed is only 0.64% [3], which severely constrains improvements in reproductive efficiency and economic return. Estrus synchronization has been shown to improve reproductive performance by increasing the twinning rate to approximately 15% [4], enhancing mating efficiency, reducing barren rates in large-scale production systems, and facilitating the application of assisted reproductive technologies such as artificial insemination and embryo transfer [5]. However, reproductive management strategies alone are insufficient to overcome the inherent genetic constraints that underlie the low fecundity of this breed. Letrozole (LE), a third-generation highly selective aromatase inhibitor, suppresses aromatase activity and blocks the conversion of androgens to estrogens. By reducing circulating estrogen levels, it decreases the negative feedback on the hypothalamic–pituitary axis, leading to increased pulsatile secretion of GnRH and subsequent elevation of FSH and LH. This endocrine modulation promotes follicular recruitment, growth, and ovulation [6]. LE has been used to induce superovulation in dairy cows and improve the reproductive performance of high-quality breeding stock [7]. Clinical studies have suggested that LE may increase ovulation and pregnancy rates, with some reports also indicating improved live birth outcomes, while maintaining a relatively low risk of multiple pregnancy [8]. A recent report on cross-bred Hamdani sheep also proved that estrous cycle hormones are key regulators of fertility [9].

In this context, Turpan Black ewes (*Ovis aries*), a native sheep breed in Turpan, Xinjiang, were used to evaluate the effects of LE administration combined with estrus synchronization on reproductive performance, including estrous response, conception rate, and twinning rate. Furthermore, beyond regulating steroid hormones, LE also modulates amino acid and lipid metabolism closely related to ovarian function. For this reason, untargeted metabolomics was applied to explore LE-induced metabolic changes. In this study, we explored the combined effects of LE and estrus synchronization on reproductive indicators. We also analyzed differential metabolites and their correlations with reproductive hormones. The present work aims to clarify the potential mechanisms of LE in regulating reproduction and provide theoretical support for improving lambing performance of Turpan Black sheep.

## 2. Materials and Methods

### 2.1. Institutional Review Board Statement

The animal study protocol was approved by the Animal Experiment Ethics Committee of Xinjiang Agricultural University (Protocol Permit Numbers: 2020032, 7 May 2020; 2020024, 20 March 2020). The study was conducted in accordance with the local legislation and institutional requirements.

### 2.2. Experimental Time and Location

This study was conducted from May to October 2025 at Huishang Ecological Pastoral Industry Co., Ltd. in Toksun County, Turpan City, Xinjiang (coordinates 87°14′05″–89°11′08″ E, 41°21′14″–43°18′11″ N).

### 2.3. Animals and Management

All experimental Turpan Black ewes were ear-tagged and raised under total confined feeding conditions. A total of 66 multiparous, non-pregnant Turpan Black ewes (Non-breeding season) were included in the study. All animals were of similar age (approximately 2.5 years), in good health, and had an average weight of approximately 35.53 ± 2.25 kg. Animals were randomly divided into two experimental groups of equal size (*n* = 33 per group), consisting of a control group and an LE-treated group. Turpan Black ewes in the LE-treated group were supplemented with letrozole at a daily dose of 0.2 mg/kg BW from Day 9 to Day 13.

### 2.4. Feeding Management

Letrozole (LE, 99% purity) was an off-white crystalline powder obtained from Shandong Senlaixi Chemical Co., Ltd., Jinan, China The powder was uniformly incorporated into 2 g of basal diet, followed by air-drying at ambient temperature. Turpan Black ewes were orally administrated with LE at a daily dose of 0.2 mg/kg BW^−1^ at 09:00 every morning for 5 days according to Rezaei et al. [10]. During administration, Turpan Black ewes were restrained, and the dough was delivered through the corner of the mouth. Their mouths were gently held until complete swallowing before releasing them. The basal total mixed ration (TMR) was provided twice daily at 10:00 and 16:30 via feeding vehicles, and the mixed diet was supplied by Huishang Ecological Animal Husbandry Co., Ltd., Toksun County, Turpan City, Xinjiang. All Turpan Black ewes had free access to feed and clean drinking water. The composition and nutritional levels of experimental diets are presented in Table 1.

### 2.5. Experimental Design

#### Estrus Synchronization Treatment

The Turpan Black ewes were fitted with progesterone-impregnated CIDR (Controlled Internal Drug Release) vaginal sponges, and the day of insertion was defined as Day 0. On Day 14, the sponges were removed, followed by intramuscular injection of 0.2 mg cloprostenol sodium (a synthetic prostaglandin F_2_α analogue) per ewe. Estrus detection and blood sampling were performed 24–48 h after sponge withdrawal, and estrus responses were recorded accordingly.

Estrus was checked daily using fertile rams fitted with anti-mating aprons. Rams were placed into ewe pens at a ram-to-ewe ratio of 1:16 for 1 h each during 09:00–10:00 and 20:00–21:00. The Turpan Black ewes that accepted mounting by teaser rams were judged to be in standing estrus and separated for artificial insemination (AI). A second AI was conducted 12 h later to improve conception outcomes.

Semen was collected from breeding rams using an artificial vagina with estrous ewes as teasers at estrus onset and 12 h thereafter. Semen collection was performed twice per insemination day with an interval of more than 4 h between two samplings. Immediately after collection, all raw semen samples were microscopically evaluated for sperm motility, concentration and morphology; only qualified semen with motility ≥ 0.7 was diluted with sheep-specific extender. Diluted semen was maintained at 22–25 °C and utilized for artificial insemination (AI) within 1 h without cryopreservation. AI, rather than natural mating, was adopted in this study for multiple scientific merits: it can eliminate confounding interference derived from variations in ram libido and sperm quality, standardize consistent insemination dose and timing across all experimental groups to improve data comparability, reduce the risk of vertical disease transmission between individuals, and generate results with practical guiding value for local large-scale sheep breeding. Each ewe received two cervical inseminations during estrus. All experimental rams were randomly allocated to the Control and LE groups to balance the baseline physiological status and semen quality of rams supplying semen for different ewe treatment groups, thereby minimizing experimental bias.

**Figure 1:**
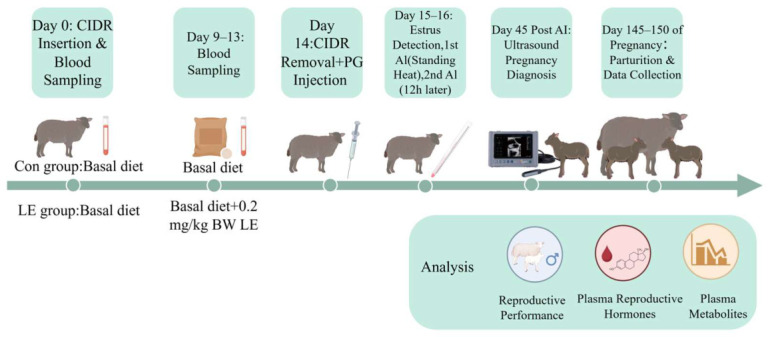
Experimental Flow Diagram.

Pregnancy was confirmed by ultrasonography 45 days after insemination; this was conducted via transabdominal B-mode ultrasonography (Model RKU101915011, Xuzhou Kaixin Electronic Equipment Co., Ltd., Xuzhou, China) on Day 45 post-insemination. The Turpan Black ewes had a gestation period of 145–150 days. After parturition, lambing rate and twinning rate were calculated based on the number of viable lambs. The total number of lambs (including stillborn and weak lambs) was also recorded, and the reproduction rate was calculated excluding stillborn and weak individuals. The detailed experimental workflow is presented in Figure 1.

### 2.6. Sample Collection and Measurements

#### 2.6.1. Feed Sample

All diets were sourced from a single batch and kept consistent across the entire trial. Representative feed samples were collected once after the adaptation period to determine moisture content and nutritional composition, and the detailed composition results are summarized in Table 1. The dry matter (DM) content of the diet was measured via oven drying at 105 °C following the Chinese national standard GB/T 6435-2014 [11]. Crude ash was determined according to GB/T 6438-2025 [12]. Crude protein was assayed using the Kjeldahl method (AOAC 990.03) [13]. Calcium was quantified by o-cresolphthalein colorimetry (AOAC 968.08) [14] and phosphorus by ammonium vanadate colorimetry (AOAC 965.17) [15]. Neutral detergent fiber (NDF) (AOAC 2002.04) [16] and acid detergent fiber (ADF) were analyzed in line with AOAC 973.18 [17].

#### 2.6.2. Plasma Sample

Six Turpan Black ewes were randomly selected from the control group and the LE group for jugular blood collection. Blood samples were collected 2 h before feeding on Day 0, Day 9, and Day 14. A total of 15 mL of blood was collected from the jugular vein into sodium heparin anticoagulant tubes and centrifuged at 3500 rpm for 15 min. The plasma supernatant was transferred into 2 mL cryovials and stored at −20 °C for analysis of plasma reproductive hormone concentrations. An additional 2 mL plasma aliquot was immediately frozen in liquid nitrogen for untargeted metabolomics analysis.

Plasma samples were thawed at 4 °C and centrifuged at 3000 rpm for 5 min to remove precipitates. Concentrations of GnRH, FSH, LH, E_2_, P_4_, and T were determined using commercial ELISA kits (Shanghai Enzyme-linked Biotechnology Co., Ltd., Shanghai, China) in accordance with the manufacturer’s instructions. Absorbance was measured at the specified wavelength using a microplate reader.

All kits complied with the manufacturer’s quality criteria, with intra-assay CV < 10% and inter-assay CV < 10%. The linear detection ranges of each kit are listed below:

GnRH: 15.62–1000 pg/mL
FSH: 1.56–100 mIU/mL
LH: 0.2–25 pg/mL
E_2_: 2.35–150 pg/mL
T: 1.715–1250 pg/mL
P_4_: 17.15–12,500 pg/mL

All detected samples were within the linear detection range of corresponding kits. The assay accuracy met the standard requirements specified by the manufacturer.

For untargeted metabolomics analysis, plasma samples were thawed at room temperature. Briefly, 100 μL of thawed plasma was transferred into an EP tube and mixed with 400 μL of 80% aqueous methanol. The mixture was fully vortexed and incubated on ice for 5 min to achieve protein precipitation. Subsequently, the sample was centrifuged at 15,000 rpm and 4 °C for 15 min. The collected supernatant was diluted with mass spectrometry-grade water to adjust the methanol concentration to 53% (*v*/*v*). This concentration was optimized to stabilize metabolites and reduce matrix interference during LC-MS detection. LC-MS detection was performed following standard protocols of the NovoMagic platform. Chromatographic separation was carried out on a C_18_ column with gradient elution. The mobile phase consisted of water containing 0.1% formic acid and acetonitrile containing 0.1% formic acid. Mass spectrometry was operated in electrospray ionization (ESI) positive ion mode, with the mass-to-charge (*m*/*z*) scanning range set from 100 to 1000. All untargeted metabolomics tests were performed by Novogene Bioinformatics Technology Co., Ltd., Beijing, China. The untargeted metabolomics analytical workflow, including LC-MS detection, multivariate statistical analysis via PLS-DA, KEGG pathway mapping, and potential biomarker identification, was performed following well-established protocols described in previous research [18].

#### 2.6.3. Formulas for Reproductive Performance Parameters


Estrus rate=(Number of estrous ewes/Total number of experimental ewes)×100%

Conception rate=(Number of pregnant ewes/Number of inseminated ewes)×100%

Lambing rate=(Total number of lambs born/Number of ewes that lambed)×100%

Twin lamb rate=(Number of ewes producing twin lambs/Number of lambed ewes)×100%

Reproduction rate=(Total number of live lambing rate/Total inseminated ewes)×100%

Note: The lambing rate and twin lamb rate were recorded. The total number of lambs born, including stillborn and weak lambs (2 in the Control group and 1 in the LE group), was recorded.

### 2.7. Data Analysis

Hormone data were sorted and collated in Microsoft Excel 2024, then analyzed via the MIXED procedure of SAS 9.4 (SAS Institute Inc., Cary, NC, USA) based on a repeated-measures design. In this mixed model, treatment group and sampling time were set as fixed effects, while individual ewe ID was defined as the random effect. Data normality was examined using the Shapiro–Wilk test, and homogeneity of variance was evaluated by Levene’s test. Post hoc multiple comparisons were performed using the Tukey–Kramer method when significant differences were detected. Estrus rate, conception rate, twinning rate and live lambing rate were analyzed by Pearson’s chi-square test in SPSS 27.0. All expected cell counts were greater than 5, and two-tailed asymptotic *p*-values were used. Since lambing rate failed to follow a normal distribution, the Mann–Whitney U test was applied for intergroup comparison. All hormone values presented in Table 2 are the overall least squares means of 6 ewes within each group across all three sampling time points (0 d, 9 d, and 14 d). *SEM* refers to the standard error of the least squares mean (standard error of LS-means) generated by the repeated measures mixed model in SAS. This *SEM* quantifies the sampling error of the overall group least squares mean, which integrates all individual hormone measurements of 6 ewes at three sampling time points (0 d, 9 d, 14 d) while correcting for repeated measurement errors within each ewe. Each group included 6 valid replicates (*n* = 6 per group). Statistical significance was set at *p* < 0.05, and *p* < 0.01 was considered highly significant.

## 3. Results

### 3.1. Effect of LE on Plasma Reproductive Hormones in Turpan Black Ewes

The effects of oral LE administration on reproductive hormone levels in Turpan Black ewes are summarized in Table 2. For E_2_, no significant intergroup difference was observed (*p* > 0.05). The time effect was highly significant (*p* < 0.01), while the group × time interaction was not significant (*p* > 0.05). For T, both group and time effects reached a highly significant level (*p* < 0.01), and their interaction showed no statistical difference. The levels of LH and P_4_ were higher in the LE treatment group. Group effect, time effect and group × time interaction were all highly significant for these two hormones (*p* < 0.01). The GnRH concentrations increased markedly in the LE group, with a highly significant group effect and group × time interaction (*p* < 0.01). The FSH presented a significant group effect and group × time interaction (*p* < 0.05), and a highly significant time effect (*p* < 0.01).

### 3.2. Effect of LE Administration on Reproductive Performance Parameters in Turpan Black Ewes

As shown in Table 3, the twinning rate was significantly higher in the LE group than in the Control group after LE administration (*p* < 0.05). Although estrus rate, conception rate, lambing rate, and reproduction rate were numerically higher in the LE group, none of these variables differed significantly between groups (*p* > 0.05).

### 3.3. Effect of Letrozole Administration on Plasma Differential Metabolites in Turpan Black Ewes

#### 3.3.1. Partial Least Squares Discriminant Analysis (PLS-DA) Model and Permutation Verification

**Figure 2:**
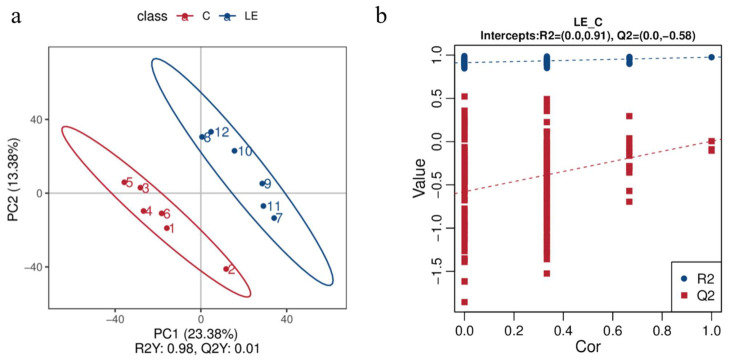
PLS-DA score plot (**a**) and Sorting Verification Diagram (**b**). Note: C represents the Control group and LE represents the LE group.

As shown in Figure 2a, the PLS-DA model was established based on combined positive and negative ion metabolomic data of the control and LE groups, with two principal components extracted. The first and second components explained 23.38% and 13.38% of the total variance, respectively. The model showed a high goodness-of-fit (R^2^Y = 0.98), while the predictive ability was extremely low (Q^2^Y = 0.01). This obvious difference indicates that the model displayed a visual grouping trend between the two groups, yet it suffered from poor predictive performance and low overall robustness. A permutation test was subsequently performed to further evaluate model reliability (Figure 2b). The intercepts of R^2^ and Q^2^ were (0.0, 0.91) and (0.0, −0.58), respectively. The negative intercept of Q^2^ verified that the PLS-DA model was not obtained from random grouping. Nevertheless, the permutation test could not offset the defects caused by the extremely low Q^2^Y value, and the model still lacked satisfactory predictive power.

#### 3.3.2. Screening and Analysis of Plasma Differential Metabolites

**Figure 3:**
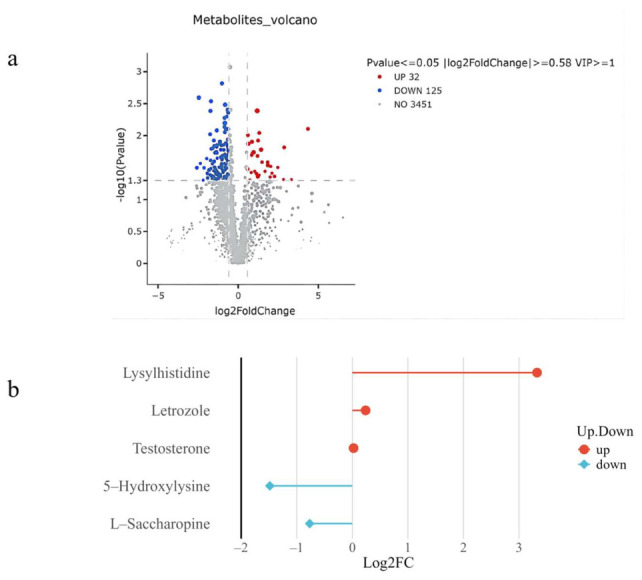
Volcano plot of differentially expressed metabolites (**a**). Stem Diagram of differentially expressed metabolites (**b**). Note: In the volcano plot, red represents upregulated differential metabolites, blue indicates downregulated differential metabolites, and gray denotes metabolites with no significant differences; the color of stem-and-leaf bars distinguishes metabolite upregulation (red) and downregulation (blue), and bar length directly reflects the value of log_2_(Fold Change); namely, longer bars mean larger fold changes. In addition, the size of dots represents the VIP value.

As shown in Figure 3a, a total of 3451 metabolites were identified in plasma samples from Turpan Black ewes in the Control and LE groups. Differential metabolites were defined by the criteria of *p* ≤ 0.05, |log_2_FoldChange| ≥ 0.58 (corresponding to fold change ≥ 1.5 or ≤0.67), and variable importance in projection (VIP) ≥ 1. Using these thresholds, 157 metabolites were identified as significantly different between groups, accounting for 4.55% of the total identified metabolites. Specifically, 32 metabolites were significantly upregulated in the LE group, representing 0.93% of the total metabolites and 20.38% of the differential metabolites. Meanwhile, 125 metabolites were significantly downregulated, representing 3.62% of the total metabolites and 79.62% of the differential metabolites. The remaining metabolites showed no significant differences between groups (*p* > 0.05). Relative to the Control group, the significantly upregulated metabolites in the LE group included lysylhistidine and Letrozole (*p* < 0.05). The significantly downregulated metabolites included L-saccharopine (*p* < 0.01), 5-hydroxylysine (*p* < 0.01), and estrone (*p* < 0.05) (Figure 3b).

#### 3.3.3. KEGG Enrichment Pathway Analysis of Plasma Differential Metabolites

**Figure 4:**
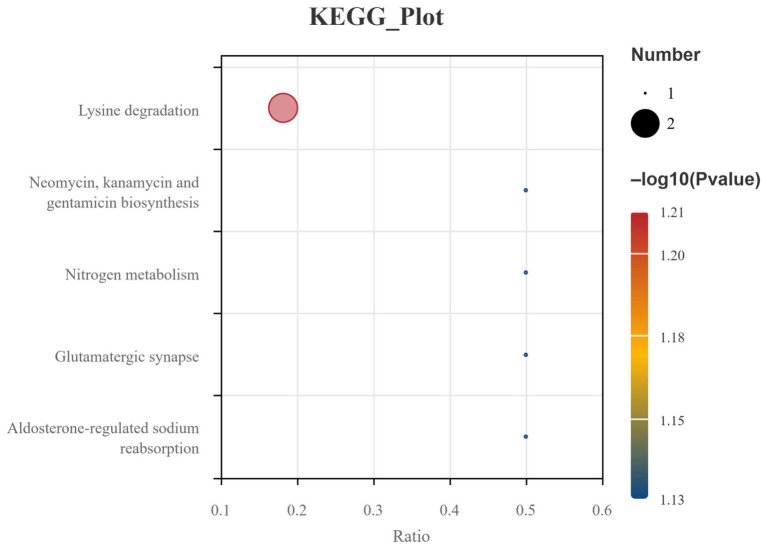
KEGG Enrichment Bubble Plot. Note: The abscissa represents enrichment ratio, indicating the proportion of differential metabolites in each pathway. Bubble size stands for the number of differential metabolites involved in the pathway, and color depth reflects the negative logarithm of enrichment significance (−log_10_(*p*-value)).

As shown in Figure 4, differentially expressed metabolites were significantly enriched in five core biological pathways: lysine degradation; neomycin, kanamycin, and gentamicin biosynthesis; nitrogen metabolism; glutamatergic synapse; and aldosterone-regulated sodium reabsorption. Pathway enrichment analysis was performed using the KEGG database. Statistical significance was assessed using the hypergeometric test, followed by Benjamini–Hochberg FDR correction for multiple comparisons. Pathways with an adjusted *p*-value (FDR) < 0.05 were considered significantly enriched. Among these pathways, lysine degradation showed the most significant enrichment (adjusted *p*-value ≈ 0.05, enrichment ratio ≈ 0.18, mapped to 2 differentially expressed metabolites). The enrichment ratios for the remaining four pathways were all 0.50, each mapping to one differentially expressed metabolite, with adjusted *p*-values ranging from approximately 0.07 to 0.075.

#### 3.3.4. Correlation Analysis Between Differential Metabolites and Reproductive Hormones

**Figure 5:**
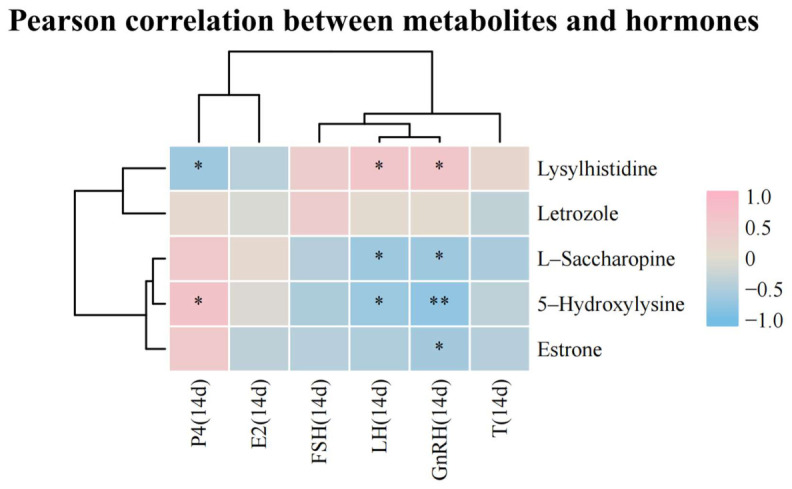
Correlation Analysis Between Plasma Differential Metabolites and Reproductive Hormones. Note: * denotes significant difference (*p* < 0.05), and ** denotes highly significant difference (*p* < 0.01).

Pearson correlation analysis was performed using the combined data from 12 Turpan Black ewes (*n* = 12; 6 in the Control group and 6 in the LE group) to investigate the associations between plasma differential metabolites and reproductive hormones. Multiple testing correction was applied using the Benjamini–Hochberg FDR method to control for false positives. As presented in Figure 5, lysylhistidine showed significant negative correlations with GnRH (14d) (*p* < 0.05), LH (14d) (*p* < 0.05), and FSH (14d) (*p* < 0.05). *L*-saccharopine was significantly positively correlated with GnRH (14d) (*p* < 0.05) and LH (14d) (*p* < 0.05). Estrone was significantly positively correlated with GnRH (14d) (*p* < 0.05). 5-hydroxylysine showed an extremely significant positive correlation with GnRH (14d) (*p* < 0.01), a significant positive correlation with LH (14d) (*p* < 0.05), and an extremely significant positive correlation with FSH (14d) (*p* < 0.01). No significant correlations were observed between the metabolites and T (14d), P_4_ (14d), or E_2_ (14d) (*p* > 0.05). It should be noted that these results only reflect correlative associations and do not establish causal relationships between metabolites and reproductive hormones.

## 4. Discussion

Letrozole inhibits aromatase activity to reduce estrogen synthesis, regulates the HPO axis and elevates gonadotropin levels, which promotes follicular growth and ovulation [19,20,21,22]. In the present study, LE administration produced a time effect on plasma T and E_2_ concentrations in Turpan Black ewes (*p* < 0.01). No significant interaction was found between treatment and time.

Previous studies have confirmed that LE does not directly bind to estrogen receptors (ER) or competitively antagonize estrogen. Reduced estrogen levels will not impair endometrial receptivity. Based on available literature, these hormonal changes may create a favorable environment for embryo implantation and development [23]. Previous studies in ruminants have demonstrated that LE treatment prolongs the dominant follicle phase and synchronizes follicular wave development [24]. Follicular growth and oocyte maturation are jointly regulated by the HPO axis and intrafollicular microenvironment [25]. Hypothalamic GnRH stimulates FSH secretion, which drives granulosa cell proliferation and estradiol production, and the resulting rise in estradiol creates a positive feedback loop that further accelerates follicular growth [26,27]. As reported previously, LE can upregulate FSHR expression and thus enhance FSH-mediated signaling to promote follicular development [28]. In the present study, dietary LE administration significantly increased plasma GnRH and LH levels, induced an upward trend in testosterone, and decreased plasma P_4_ concentration (*p* < 0.01) in Turpan Black ewes. These results are consistent with the known pharmacological effects of LE. The elevated GnRH and LH concentrations indicate that LE weakens estrogen-induced negative feedback on the HPO axis and restores pulsatile gonadotropin secretion. The rising testosterone level is associated with inhibited aromatase activity and reduced conversion of androgens to estrogens.

No significant intergroup differences were observed in T and E_2_ concentrations. The following interpretation is proposed as a hypothesis: The lack of statistical difference may be related to the sampling time. Circulating LE concentration may have decreased at sampling, and gonadotropins rebounded after the relief of negative feedback. Elevated gonadotropins may have reinitiated estrogen synthesis in follicles, while compensatory peripheral androgen metabolism pathways may have also maintained steady E_2_ levels. The above factors may collectively account for the non-significant difference in plasma E_2_. The decreased P_4_ concentration (*p* < 0.01) suggests that experimental Turpan Black ewes were in the early follicular phase with incomplete corpus luteum function. This physiological state matches the synchronized follicular development after estrus synchronization.

FSH acts directly on the ovary to drive follicle recruitment, growth and maturation. Elevated FSH levels help form a favorable endocrine environment for synchronous follicular developmen [29]. In the present study, estrus rate, conception rate and lambing rate rose in LE-treated Turpan Black ewes, though these changes were not statistically significant. Such trends may be associated with the regulatory effect of LE on follicle and oocyte development. LE suppresses aromatase activity to lower estrogen concentrations and reduce negative feedback on the HPO axis. This process increases the secretion of FSH and LH. Rising FSH facilitates follicular development, while elevated LH triggers ovulation. According to published literature, these hormonal changes may potentially affect oocyte quality, fertilization and embryo implantation. We speculate that limited sample size, individual differences in ovulation performance, and technical factors during estrus detection and artificial insemination may account for the non-significant results above. These explanations remain hypothetical. Further studies with larger sample sizes and improved monitoring methods are needed to confirm the observed trends.

Untargeted metabolomics combined with Pearson correlation analysis revealed significant changes in three key metabolites in Turpan Black ewes after dietary LE supplementation. L-saccharopine and 5-hydroxylysine were decreased (*p* < 0.01), while lysylhistidine was increased (*p* < 0.05). These differential metabolites were mainly enriched in the lysine degradation pathway (map00310).

Lysine is an essential amino acid, and it is primarily catabolized via the saccharopine pathway. L-saccharopine acts as a core intermediate in this pathway and finally enters the tricarboxylic acid cycle for energy supply [30,31]. Based on the altered level of L-saccharopine, we hypothesize that LE may change lysine metabolic flux. This is a speculative inference: lysine may be less utilized for catabolic energy production and more directed toward anabolic protein synthesis. LE inhibits aromatase activity to reduce peripheral estrogen levels and relieve estrogen-mediated negative feedback on the HPO axis. This further elevates the secretion of GnRH, LH and FSH and promotes follicular development and granulosa cell proliferation [23,24]. Follicular growth increases the demand for amino acids. We speculate that lysine may be preferentially used for protein synthesis in follicles rather than catabolized via the saccharopine pathway. This potential link between lysine metabolism and ovarian function remains to be further verified. 5-hydroxylysine participates in the saccharopine pathway and serves as a precursor for collagen synthesis related to extracellular matrix remodeling. Its downregulation may reflect altered enzyme activity or metabolic substrate redistribution [32,33]. Lysylhistidine is a dipeptide derived from lysine and histidine. Its upregulation may be associated with altered protein turnover or peptidase activity [34]. In summary, LE can modulate plasma lysine metabolism in Turpan Black ewes. It mainly affects the saccharopine pathway, lysine hydroxylation process and dipeptide abundance.

A positive correlation was observed between lysylhistidine and GnRH as well as LH. This metabolite also presented a negative correlation with P_4_ (*p* < 0.05). Histidine-containing dipeptides act as precursors for histamine synthesis. Studies have confirmed that hypothalamic histaminergic neurons modulate pulsatile GnRH secretion [35]. Considering the structural characteristics of lysylhistidine, we propose the following as a hypothesis: this dipeptide may potentially affect GnRH secretion by regulating histaminergic neuronal activity. No direct evidence currently supports this linkage. Ye et al. found that hypothalamic anserine and L-histidine levels rose gradually during sexual maturation in female goats [36]. The two metabolites belong to histidine-related substances, while they differ from lysylhistidine detected in our study. Combined with published literature, we note that elevated lysylhistidine is associated with HPO axis-related hormonal changes under LE treatment. This is merely an observed correlative relationship, and further studies are needed to explore the underlying mechanisms. Estrone was negatively correlated with GnRH, which matches the classical estrogen negative feedback on GnRH secretion. LE suppresses aromatase to reduce estrogen production and relieve feedback inhibition. Remaining plasma estrone still maintains partial inhibitory effects. This pattern may indicate partially relieved negative feedback and stable endocrine balance, which explains the increased GnRH and unchanged E_2_ levels. L-saccharopine was negatively correlated with GnRH and LH. Its downregulation may reflect reduced lysine catabolism and increased lysine storage in vivo [37]. Lysine participates in protein synthesis and epigenetic regulation. Inhibition of the saccharopine pathway could potentially shift lysine toward histone modification. This metabolic alteration may be associated with reproductive regulation, and relevant mechanisms need to be validated in future work. 5-hydroxylysine was negatively correlated with GnRH and LH, and positively correlated with P4. It is a key modification product of collagen. Its variation may indirectly influence ovarian function by regulating the extracellular matrix and follicular structure [38].

**Figure 6:**
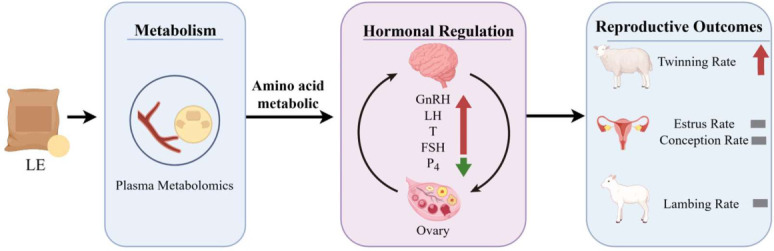
Proposed mechanism of letrozole (LE) affecting reproductive performance in Turpan Black ewes. Black straight arrows show the unidirectional transmission of metabolic and reproductive regulatory signals induced by LE. Circular black double-headed curved arrows represent the feedback regulatory loop between the hypothalamic-pituitary gland and ovary. The red upward arrow marks the upregulation of GnRH, LH, T and FSH, while the green downward arrow indicates the downregulation of P_4_. The vertical red upward arrow adjacent to Twinning Rate reflects a marked rise in twinning rate, and gray horizontal bars indicate that estrus rate, conception rate and lambing rate did not differ significantly after LE supplementation.

In summary, LE may affect reproductive physiology in Turpan Black ewes via two potential pathways. The first pathway is well supported: LE directly inhibits aromatase activity and relieves estrogen-mediated negative feedback on the HPO axis. The second pathway is proposed based on metabolomic and correlation results. LE may remodel the lysine metabolic network. Combined with correlative data, we hypothesize that changes in lysine metabolism could potentially influence GnRH and LH pulsatile secretion by regulating histaminergic neurotransmitter synthesis, protein turnover and epigenetic modification. Taken together, the changes in hormonal profiles and lysine metabolism may jointly be associated with follicular development in LE-treated ewes. Further targeted experiments are required to verify the underlying causal relationships (Figure 6).

## 5. Conclusions

Under the experimental conditions of this study, LE administration changed circulating reproductive hormones and lysine-associated metabolites in Turpan Black ewes. Lysylhistidine and L-saccharopine, as key differential metabolites, were concentrated in lysine metabolism pathways. Estrus, conception and lambing rates showed rising trends without statistical significance. These results offer a novel reference and theoretical support for optimizing the reproductive performance and twinning rates of Turpan Black ewes.

## 6. Limitations

First, body condition score (BCS), a critical indicator reflecting energy reserve and nutritional status that strongly influences ewe reproductive performance, was not recorded in the present experiment. Although all experimental ewes exhibited a consistent initial body weight (35.53 ± 2.25 kg), body weight is less sensitive than BCS in evaluating body fat variation and energy balance status. The lack of BCS data limits the interpretation of individual differences in reproductive responses to LE treatment. Future studies should incorporate systematic BCS evaluation to better clarify the associations among LE intervention, nutritional status, and reproductive performance.

Second, the LH sampling strategy used in this study has inherent limitations. High-frequency serial sampling every 15–30 min for 2–3 h is optimal for characterizing LH pulse dynamics and distinguishing sustained hormonal elevation from accidental capture of sporadic LH peaks. However, intensive short-interval venipuncture was unfeasible here, as it would have induced severe stress and disrupted endogenous LH secretion, while adequate labor support for continuous sampling was unavailable. We adopted 1 h interval sampling with 2–3 repeated collections per ewe to reduce detection bias compared with single-point sampling. Nevertheless, we cannot fully rule out the possibility that elevated LH levels in the LE group result from random pulse capture rather than stable treatment-induced hormonal changes. Therefore, the current LH results are interpreted conservatively. Further studies with dense serial sampling are required to precisely reveal the regulatory effects of LE on LH secretion patterns.

## Figures and Tables

**Table 1:** Diet Formula Composition and Nutrient Level (DM basis) (%).

Ingredients	Content	Nutrient Levels ^②^	Content
Whole corn silage	48.55	DM	53.81
Corn	14.85	CP	9.85
Wheat bran	8.56	EE	2.35
Soybean meal	5.28	Ash	5.52
Cotton seed meal	2.58	NDF	46.71
Sorghum stalks	13.86	ADF	27.55
Bioactive peptide	2.5	Ca	0.32
NaHCO_3_	0.51	P	0.24
NaCl	0.31	ME/(MJ·kg^−1^)	8.05
Premix ^①^	3		
Total	100		

Note: ^①^ Per kilogram of premix: Vitamin A 150,000 IU, Vitamin D_3_ 8000 IU, Vitamin E 1000 IU, Vitamin K_3_ 10 mg, Copper 150 mg, Zinc 600 mg, Manganese 450 mg, Iron 600 mg, Iodine 15 mg, Cobalt 5 mg, Selenium 5 mg. ^②^ All nutrient levels are based on actual measured values; metabolizable energy is a calculated value.

**Table 2:** Effect of Letrozole Administration on Plasma Reproductive Hormone Levels in Turpan Black Ewes (*n* = 6).

Items	Group		*SEM*	*p*-Value		
	Control Group	LE Group		Group	Time	Group × Time
E_2_ (pg/mL)	12.78	12.96	0.16	0.58	<0.01	0.54
T (pg/mL)	7.62 ^B^	8.04 ^A^	0.07	0.01	<0.01	0.63
LH (ng/mL)	0.60 ^B^	0.89 ^A^	0.01	<0.01	<0.01	<0.01
GnRH (pg/mL)	41.92 ^B^	61.29 ^A^	0.68	<0.01	0.25	<0.01
FSH (mIU/mL)	16.43 ^b^	16.70 ^a^	0.06	<0.03	<0.01	0.03
P_4_ (ng/mL)	2.19 ^A^	2.03 ^B^	0.01	<0.01	<0.01	<0.01

Note: The absence of a letter indicates no significant difference (*p* > 0.05); different lowercase letters indicate significant differences (*p* < 0.05); different uppercase letters indicate highly significant differences (*p* < 0.01).

**Table 3:** Effect of LE Administration on Production Performance Parameters of Turpan Black Ewes.

Items	Groups		*p*-Value
	Control Group	LE Group	
Estrous rate	72.73% (24/33)	75.76% (25/33)	0.78
Conception rate on 45 d	72.73% (24/33)	78.79% (26/33)	0.57
Lambing rate	104.17% (25/24)	119.23% (31/26)	0.17
Twin lambing rate	0 (0/24) ^b^	15.38% (4/26) ^a^	0.04
Reproduction rate	69.70% (23/33)	87.88% (29/33)	0.07

Note: In the data tables, different lowercase letters indicate a significant difference (*p* < 0.05); the absence of a letter indicates no significant difference (*p* > 0.05).

## Data Availability

All relevant data supporting the findings of this study are included within this article.

## Abbreviations

ADF	Acid Detergent Fiber
BW	Body Weight
CP	Crude Protein
CIDR	Controlled Internal Drug Release
DM	Dry Matter
AI	Artificial insemination
E_2_	Estradiol
ER	Estrogen receptors
FSH	Follicle-Stimulating Hormone
FDR	False discovery rate
GnRH	Gonadotropin-Releasing Hormone
HPO axis	Hypothalamic–pituitary–ovarian axis
LE	Letrozole
LH	Luteinizing Hormone
SEM	Standard error of the means
TMR	Total Mixed Ration
T	Testosterone
P_4_	Progesterone

## References

[1] Ruozhanguoli R. (2013). A Preliminary Study on Germplasm Characteristics of Turpan Black Sheep. Master’s Thesis.

[2] Hainimuguli A. (2008). Protection and development strategies of Turpan black sheep breed. Chin. Anim. Husb. Vet. Med..

[3] Qi X., Wang Z., Zhang P. (2015). Turpan black sheep’s protection use of germplasm resource in Toksun County. Hans. J. Agric. Sci..

[4] Abulaike A., Saimi A., Mailikere W. (2015). Application of Efficient Breeding Technology for Turpan Black Sheep. Xinjiang Anim. Husb..

[5] Xayalath S., Novotni-Danko G., Rátky J. (2022). The Role of Estrous Synchronization and Artificial Insemination in Improving the Reproductive Performance of Moo Lath Gilts. Agriculture.

[6] Romanski P.A., Shah N.J., Bortoletto P., Rosenwaks Z., Schattman G. (2020). Effect of Follicle Size at Trigger in GnRH Antagonist Plus Letrozole IVF Cycles on Oocyte Quality Outcomes. Fertil. Steril..

[7] Zwiefelhofer E.M., Lillico W., Adams G.P. (2022). Development of a letrozole-based synchronization protocol for fixed-time artificial insemination in beef cattle. Anim. Reprod. Sci..

[8] Miller R.A.K., Pinheiro M.G. (2025). Comparative Outcomes of Letrozole Versus Clomiphene Citrate for Ovulation Induction in Patients With PCOS: Systematic Review and Meta-Analysis. JBRA Assist. Reprod..

[9] Turgut A.O., Koca D. (2024). Serum Anti-Müllerian hormone levels during estrus and diestrus phases of the estrous cycle and its possible effect on fertility in cross-bred Hamdani sheep. Pak. Vet. J..

[10] Rezaei A., Vaziry A., Farshad A., Farzinpour A., Rostamzadeh J. (2020). Effects of letrozole administration on growth and reproductive performance in Markhoz goat bucklings. Theriogenology.

[11] (2014). Determination of Moisture in Feedstuffs.

[12] (2025). Determination of Crude Ash in Feedstuffs.

[13] AOAC International (2006). AOAC Official Method 990.03: Crude Protein in Animal Feed, Combustion Method. Official Methods of Analysis.

[14] AOAC International (2005). AOAC Official Method 968.08: Calcium in Feeds, o-Cresolphthalein Colorimetric Method. Official Methods of Analysis.

[15] AOAC International (2005). AOAC Official Method 965.17: Phosphorus in Feeds, Ammonium Vanadate Colorimetric Method. Official Methods of Analysis.

[16] AOAC International (2006). AOAC Official Method 2002.04: Amylase-Treated Neutral Detergent Fiber in Feeds. Official Methods of Analysis.

[17] AOAC International (2006). AOAC Official Method 973.18: Acid Detergent Fiber and Lignin in Animal Feeds. Official Methods of Analysis.

[18] Shah A.M., Wang Z.S., Hu R., Peng Q., Zou H., Wang L., Xue B. (2024). Discovery of the enrichment pathways and biomarkers using metabolomics techniques in unilateral and bilateral castration in yellow cattle. Pak. Vet. J..

[19] Meng Q.L. (2019). Efficacy and safety of letrozole in the treatment of recurrent endometriosis. Clin. Med. Res. Pract..

[20] Han J. (2020). Clinical efficacy of letrozole combined with different regimens in patients with polycystic ovary syndrome. J. Changzhi Med. Coll..

[21] Qu H.G., Wen T.F., Zhang X.J. (2020). Clinical observation on ovulation induction effects of letrozole and clomiphene citrate in infertile patients with polycystic ovary syndrome. Matern. Child Health Care China.

[22] Ding Y.Y., Si J.G., Li D.Y. (2023). Effects of Nuangong Yunzi Capsules combined with letrozole on endometrium and sex hormones in patients with ovulatory dysfunction infertility. Northwest Pharm. J..

[23] Mo G.H., Hong G.Z., Tang Z.X. (2024). Clinical effect of letrozole in ovulation induction treatment of infertile patients with polycystic ovary syndrome. Women Child Health J..

[24] Yapura M., Mapletoft R., Pierson R., Singh J., Adams G. (2016). Synchronization of ovulation in cattle with an aromatase inhibitor–based protocol. Theriogenology.

[25] Shabankareh H.K., Sarsaifi K., Mehrannia T. (2011). In vitro maturation of ovine oocytes using different maturation media: Effect of human menopausal serum. J. Assist. Reprod. Genet..

[26] Lambalk C.B. (2023). The enigma of the gonadotropin-releasing hormone pulse frequency governing individual secretion of luteinizing hormone and follicle-stimulating hormone. F&S Rep..

[27] Lee S.Y., Kang Y.J., Kwon J., Nishi Y., Yanase T., Lee K.-A., Koong M.K. (2020). miR-4463 regulates aromatase expression and activity for 17β-estradiol synthesis in response to follicle-stimulating hormone. Clin. Exp. Reprod. Med..

[28] Kasuga-Yamashita F., Baba T., Nagao S., Fujibe Y., Morishita M., Kuno Y., Mariya T., Honnma H., Endo T., Kiya T. (2022). Letrozole increases preantral follicle growth and decreases estradiol production without impairing follicle survival. J. Ovarian Res..

[29] Kivrak M.B., Corum O., Alkan H., Atik O., Aydin I., Uney K. (2021). The pharmacokinetics of letrozole and its effect on gonadotropins in anestrous ewes. Theriogenology.

[30] Arruda P., Barreto P. (2020). Lysine Catabolism Through the Saccharopine Pathway: Enzymes and Intermediates Involved in Plant Responses to Abiotic and Biotic Stress. Front. Plant Sci..

[31] Leandro J., Houten M.S. (2020). The lysine degradation pathway: Subcellular compartmentalization and enzyme deficiencies. Mol. Genet. Metab..

[32] Hara R., Yamagata K., Miyake R., Kawabata H., Uehara H., Kino K. (2017). Discovery of Lysine Hydroxylases in the Clavaminic Acid Synthase-Like Superfamily for Efficient Hydroxylysine Bioproduction. Appl. Environ. Microbiol..

[33] Marco D.M., Rai R.S., Scietti L., Mattoteia D., Liberi S., Moroni E., Pinnola A., Vetrano A., Iacobucci C., Santambrogio C. (2025). Molecular structure and enzymatic mechanism of the human collagen hydroxylysine galactosyltransferase GLT25D1/COLGALT1. Nat. Commun..

[34] Kofoed C., Wu S., Sørensen K.K., Treiberg T., Arnsdorf J., Bjørn S.P., Jensen T.L., Voldborg B.G., Thygesen M.B., Jensen K.J. (2022). Highly Selective Lysine Acylation in Proteins Using a Lys-His Tag Sequence. Chem. Eur. J..

[35] Kjaer A., Larsen P.J., Knigge U., Warberg J. (1995). Dehydration stimulates hypothalamic gene expression of histamine synthesis enzyme: Importance for neuroendocrine regulation of vasopressin and oxytocin secretion. Endocrinology.

[36] Ye J., Yan X., Zhang W., Lu J., Xu S., Li X., Qin P., Gong X., Liu Y., Ling Y. (2023). Integrative proteomic and phosphoproteomic analysis in the female goat hypothalamus to study the onset of puberty. BMC Genom..

[37] Bottino C., Peserico A., Simone C., Caretti G. (2020). SMYD3: An Oncogenic Driver Targeting Epigenetic Regulation and Signaling Pathways. Cancers.

[38] Nagyová E., Němcová L., Camaioni A. (2021). Cumulus Extracellular Matrix Is an Important Part of Oocyte Microenvironment in Ovarian Follicles: Its Remodeling and Proteolytic Degradation. Int. J. Mol. Sci..

